# Alginate Microbeads Containing Halloysite and Layered Double Hydroxide as Efficient Carriers of Natural Antimicrobials

**DOI:** 10.3390/nano14020232

**Published:** 2024-01-21

**Authors:** Gianluca Viscusi, Elisa Boccalon, Elena Lamberti, Morena Nocchetti, Giuliana Gorrasi

**Affiliations:** 1 Department of Industrial Engineering, University of Salerno, Via Giovanni Paolo II 132, 84084 Fisciano, Italy; gviscusi@unisa.it (G.V.); ellamberti@unisa.it (E.L.); 2 Department of Chemistry, Biology and Biotechnology, University of Perugia, Via dell’ Elce di Sotto 8, 06123 Perugia, Italy; elisa.boccalon@unipg.it; 3 Department of Pharmaceutical Sciences, University of Perugia, Via del Liceo 1, 06123 Perugia, Italy; morena.nocchetti@unipg.it

**Keywords:** microencapsulation, halloysite, layered double hydroxide, drug carrier, grapefruit seed oil

## Abstract

The present paper describes the preparation and characterization of novel microbeads from alginate filled with nanoclay such as halloysite nanotubes (HNTs). HNTs were used as support for the growth of layered double hydroxide (LDH) crystals producing a flower-like structure (HNT@LDH). Such nanofiller was loaded with grapefruit seed oil (GO), an active compound with antimicrobial activity, up to 50% wt. For comparison, the beads were also loaded with HNT and LDH separately, and filled with the same amount of GO. The characterization of the filler was performed using XRD and ATR spectroscopy. The beads were analyzed through XRD, TGA, ATR and SEM. The functional properties of the beads, as nanocarriers of the active compound, were investigated using UV-vis spectroscopy. The release kinetics were recorded and modelled as a function of the structural characteristics of the nanofiller.

## 1. Introduction

Microencapsulation is an efficient drug delivery technique that offers numerous benefits over direct intake or conventional delivery systems; these advantages include control of the release rate, protection of the active ingredient from degradation, and targeted delivery [1,2]. Microencapsulation has not only proven to be successful in pharmaceuticals, medicines and cosmetics but also in the food industry, where, in addition to controlling the release rate of antimicrobials and their targeted dispensing, it can also be used to absorb harmful substances and odours and prolongate the storage of volatile compounds, as well as to provide information on the quality of the item through a ‘smart’ response [3]. Synthetic polymers like polyesters and polyamides provide an excellent matrix to ensure microencapsulation, but natural biodegradable polymers such as gelatine, cellulose, starch, chitosan, and alginate are more appealing due to their biocompatibility, lack of toxicity and hydrophilicity [4]. Among polysaccharide-based polymers, alginates have become popular thanks to their easy processability, low cytotoxicity and easy gelation in the presence of divalent cations [5]. Over the years, alginate-based products have been experimented for different applications, ranging from biotechnology to pharmaceuticals (e.g., wound dressings, bone regeneration, protein delivery, oral drug delivery, controlled release systems) as well as cosmetic and food industries. The FDA (US Food and Drug Administration) [6], the EFSA (European Food Safety Authority) [7] and the Joint FAO/WHO Expert Committee on Food Additives (JECFA) have authorized the use of alginate in food preparations and packaging and have listed it among the substances generally recognized as safe for consumers and the environment. Thus, alginate is today commonly found as a thickener, stabilizer, texturizer, firming agent, and additive in many food preparations and in the fabrication of edible films or coatings, as well as in tablets and microspheres [8]. Alginate microspheres can deliver a wide variety of active ingredients for different purposes. However, to meet the continuing demand for natural alternatives to chemical additives in the food industry, a current trend to prevent food spoilage is to use plant extracts, such as essential oils [9,10,11,12,13]. Essential oils possess recognized antioxidant, antimicrobial and anti-inflammatory properties and can be extracted from different parts of plants (i.e., flowers, seeds, leaves, bark, wood, fruit peel and roots). Citrus derivatives, in particular, have promising market potential, as citrus species represent the largest portion of fruit produced worldwide [14]. Often added in preparations as fragrance or flavouring compounds, recently, the use of citrus by-products has been boosted thanks to their bioactivity [15,16]. Citrus derivatives are used as natural preservatives due to their biological activities, including antimicrobial and antioxidant effects. The presence of terpenes, flavonoids, carotenes and coumarins is thought to be responsible for the strong antioxidative and antimicrobial activities [9,10,11,17]. Grapefruit (*Citrus paradisi* Macfad.), for instance, is a source of phenolic and terpenic compounds with high antimicrobial properties. Grapefruit essential oil (GO) is commonly extracted from the peel of the fruit, but it can also be derived from the seeds and the pulp. The seed extract is an effective broad-spectrum bactericide, fungicide, antiviral and antiparasitic with mild inhibitory activity on some yeast strains [18]. Recent studies have also proved that GO seed extract can offer a viable solution to treat multidrug-resistant bacteria [19,20,21,22]. The incorporation of GO in alginate beads protects it from degradation and ensures its modulated release, but some limitations include the loss of active ingredients during the microbead preparation and the structural instability of the polymeric matrix [23]. One way to overcome these limitations is to reinforce the polymer with a filler and load the filler with GO so that the active ingredient is released in an even more controlled manner. In this work, we report a comparison between the loading and release properties of alginate beads containing GO and alginate beads prepared with different fillers in which GO was incorporated. The fillers tested were as follows: halloysite (HNT), layered double hydroxide (LDH) and a combination of the latter with a filler consisting of halloysite nanotubes decorated with LDH platelets (HNT@LDH). Halloysite is an aluminosilicate clay mineral with the structural formula Al_2_(OH)_4_Si_2_O_5_·nH_2_O, arranged in nanotubes of about 0.5–2 μm in length [24,25,26,27]. LDH is a layered solid with the formula [M(II)_1−x_M(III)_x_(OH)_2_] (A^n−^)_x/n_·mH_2_O, where M(II) and M(III) are divalent and trivalent cations, respectively, and A^n−^ is the intercalating anion [28,29,30,31]. HNT and LDHs are widely used as carriers and supports for active ingredients in the pharmaceutical and biomedical fields, as well as in the food packaging industry [32,33]; however, their combination has rarely been explored [34,35,36]. This work aimed to verify how the joint use of the two fillers leads to an optimization of the sustained release capacity and improves the properties of the alginate microsphere. Alginate was chosen since it is a natural polysaccharide extracted from brown seaweed [37]; its gelation property allows for encapsulating various substances through the formation of an egg-box structure [38,39,40]. This polymer has many advantages, such as availability, low cost, nontoxicity, biocompatibility, and biodegradability, and it has been applied in different applications over the last decade, including pharmaceuticals [41], food [42] and increasingly as coagulants and sorbents for dye removal [43,44].

## 2. Materials and Methods

### 2.1. Materials

Halloysite nanotubes, sodium alginate, calcium chloride, Mg(NO_3_)_2_⋅6H_2_O and Al(NO_3_)_3_⋅9H_2_O were supplied by Sigma Aldrich Chemicals (St. Louis, MO, USA). NaOH is an Alfa Aesar product (Haverhill, MA, USA). Grapefruit seed oil (GO), 100% pure cold-pressed, was purchased from Organic Herbal Essence. Ethanol was purchased from VWR Chemicals (Suwanee, GA, USA) (96%).

### 2.2. Synthesis of HNT@LDH

The filler was prepared according to the mechanochemical method previously described [34]. Specifically, LDH was synthesized by milling 826 mg of Mg(NO_3_)_2_·6H_2_O, 596 mg of Al(NO_3_)_3_·9H_2_O and 388 mg of NaOH with 800 mg of HNT in a planetary ball mill for 1 h at 30 Hz frequency. The filler was washed once with water and twice with acetone. The final product, HNT@LDH, is a composite filler composed of HNT covered by LDH nanocrystals in nitrate form in 1:0.5 HNT:LDH weight ratio. The LDH composition is as follows: [Mg_0.67_Al_0.33_(OH)_2_](NO_3_)_0.33_ 0.2·H_2_O. Ball milling was performed through a Retsch (Haan, Germany) MM200 ball mill (capacity 10 mL) provided with zirconia spheres with diameters of 10 mm. In total, 60% of the grinding jar (operating at 30 Hz) volume was filled with grinding balls, NaOH, salt powders and HNT. During the grinding, powder samples were taken at 30 and 60 min.

### 2.3. Preparation of Nanohybrids

To prepare the nanohybrids, grapefruit seed oil was added to HNT, LDH and HNT@LDH at a fixed ratio of 50:50 *w*/*w*. The hybrid loading GO mixture was then transferred into a vacuum jar connected to a vacuum pump. After vacuuming for 30 min to remove the air, air was slowly let in the vacuum jar until atmospheric pressure was reached. The mixture was kept at room temperature and ambient pressure for 10 min, allowing the grapefruit seed oil to be loaded into inorganic materials through capillary force. This procedure was repeated three times to increase the loading efficiency. The resulting nanohybrids were labelled as LDH + GO, HNT + GO and HNT@LDH + GO.

### 2.4. Preparation of Alginate Microbeads

**Figure 1:**
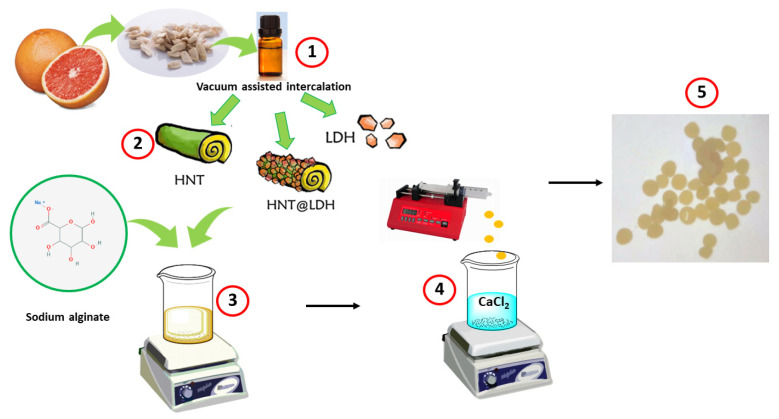
Schematization of alginate bead preparation through the following steps: (1) extraction of GO from grapefruit; (2) encapsulation of GO in the inorganic fillers: HNT, LDH and the hybrid HNT@LDH; (3) mixing of sodium alginate and LDH + GO, HNT + GO or HNT@LDH + GO; (4) dripping of solution into CaCl_2_ and occurrence of gelation; (5) drying of beads.

To prepare the beads, alginate (0.81 g) was dissolved in water (30 mL) and stirred at 100 °C for 3 h. LDH + GO, HNT + GO and HNT@LDH + GO were separately added to the solution (5% wt polymer). The solution was then added dropwise to a 1.5% wt CaCl_2_ solution to obtain the beads. To achieve complete gelation, the beads were kept at 4 °C for 24 h. Afterwards, the beads were removed from the CaCl_2_ solution, washed with distilled water and airdried for 72 h. Pure alginate beads were also made in the same way. Figure 1 depicts a schematization of the gelation process, along with a picture of the alginate beads as an example.

### 2.5. Techniques

The composition of grapefruit seed oil was determined using a GC-MS (Thermo Fischer Scientific, Waltham, MA, USA) with a TraceGOLD™ TG-WaxMS GC capillary column (0.25 µm × 0.25 mm × 30 m), heated from 50 to 250 °C at a rate of 6 °C/min. Injector and detector temperatures were set at 200 and 250 °C, respectively. Helium was used as a carrier gas with a column flow of 1 mL/min, split flow of 50 mL/min and split ratio of 50:1. The injection volume was 1 μL, and the FID was set at a temperature of 250 °C. The identification of essential oil compounds was performed by analyzing the mass spectra of each component and comparing them with reference spectra.

The diameter of the beads was evaluated by analyzing digital images. Twenty beads were analyzed for each condition. The beads’ diameters were obtained from recorded photographs and are expressed as the mean ± standard deviation.

X-ray diffraction (XRD) analyses were performed on the fillers in powder mixed with GO, and on the beads, using a Malvern Panalytical X′Pert PRO MPD diffractometer (Almelo, The Netherlands) operating at 40 kV and 40 mA, with a step size of 0.0334° and step scan of 2θ per s, using Cu Kα source radiation and an X’Celerator detector by Malvern Panalytical. The data were elaborated via the X′Pert HighScore software version 2.0

Attenuated total reflection (ATR) investigations were carried out with a Shimadzu IR-8000 spectrophotometer. The spectral range collected was 400 to 4000 cm^−1^, with a spectral resolution of 4 cm^−1^, acquiring 100 scans.

The morphology of the HNT, HNT@LDH and beads was investigated with a scanning electron microscope (FE-SEM, FEG LEO 1525, Zeiss Group Oberkochen, Baden-Württemberg, Germany). Measurements were performed with an acceleration voltage of 7 kV and a working distance of 9 mm using an In-lens detector. All samples were coated with a thin layer of chromium (8 nm) before examination. The elemental mapping of samples was carried out by using energy-dispersive X-ray spectroscopy (EDX) supported by a field emission scanning electron microscope. Magnesium, aluminium and silicon elemental analyses were carried out by using inductively coupled plasma-optical emission spectrometers (ICP-OES) (Varian, Inc., Palo Alto, CA, USA, 710-ES series). The solids were dissolved in the smallest quantity of HF and HNO_3_ concentrated solution and diluted with deionized water.

Thermogravimetric analysis (TGA) was carried out in a nitrogen atmosphere with a Mettler TC-10 apparatus (Billerica, MA, USA) from 25 to 700 °C, at a heating rate of 10 °C/min.

The amount of GO loaded into the different inorganic carriers was determined according to Equation (1) [45]:

(1) $$X=\frac{R_{\mathrm{mix}}-R_{H}}{R_{b}-R_{H}}$$

where R_mix_ is the weight loss percentage of the mixture, and R_H_ and R_b_ are the weight loss percentages of the carrier and loaded compound, respectively. This equation assumes that the total weight loss of the single component is not affected by the other components present in the mixture.

The antioxidant activity was tested against the 2,2-diphenyl-1-picrylhydrazyl radical (DPPH). Briefly, 50 mg of each sample was mixed with DPPH methanolic (4 mL, 0.0625 mM) solution. The mixtures were then left in the dark to allow the reaction to take place for 30 min. The radical scavenging was examined over time (from 5 to 30 min) along with the optical density at 517 nm using a spectrophotometer (UV-2401 PC Shimadzu, Kyoto, Japan). The antioxidant activity (AA%) was calculated using the following formula (Equation (2)) [46]:

(2) $$\mathrm{AA}\;\left(\%\right)=\left(1-\frac{A_{t}}{A_{0}}\right)\times 100$$

where A_0_ is the original absorbance of DPPH, and A_t_ is the absorbance of the DPPH at a given measuring time. By comparison, the radical scavenging activity of GO was evaluated using the same procedure by mixing 100 µL of GO with 3.9 mL of DPPH solution.

To analyze the release kinetics of GO, 200 mg of beads were placed into 10 mL of EtOH/water 50/50 *v*/*v* and stirred at 100 rpm in an orbital shaker (VDRL MOD. 711+ Asal S.r.l., Cernusco sul Naviglio, Italy). At fixed time intervals, the release medium was withdrawn and analyzed using the Shimadzu UV-2401 PC spectrophotometer. The considered band of linoleic acid at 281 nm was used for the UV-Vis analysis.

## 3. Results

### 3.1. Chemical Composition of GO

**Figure 2:**
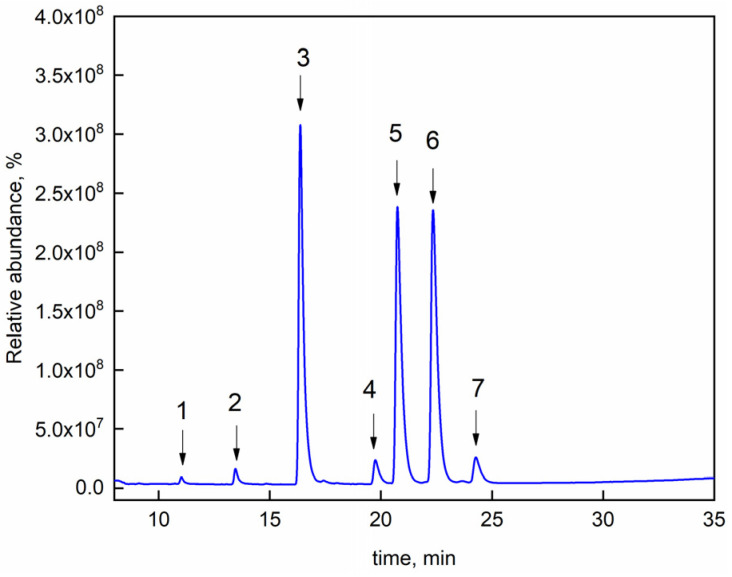
Gas chromatograph spectrum of grapefruit seed oil.

Grapefruit seed oil, like all natural essential oils, is a complex mixture of several organic compounds [47]. Prior to performing UV-vis analysis, the grapefruit seed oil composition was determined by carrying out a GC-MS analysis. The GC spectrum is reported in Figure 2. Seven main constituents were identified, as reported in Table 1.

### 3.2. XRD and FTIR Analyses

**Figure 3:**
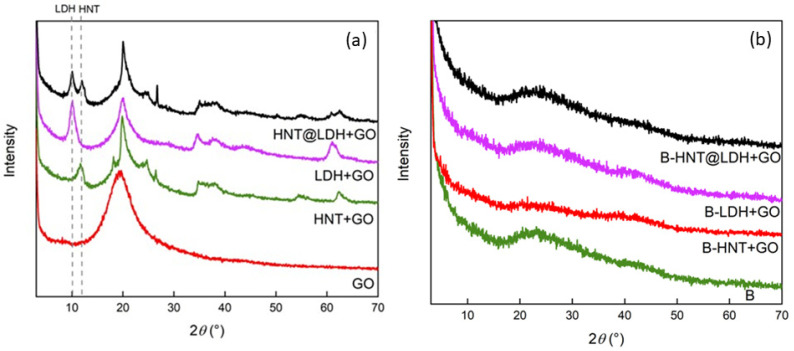
XRD patterns of GO, HNT + GO, LDH + GO, HNT@LDH + GO (**a**) and the indicated alginate beads (**b**).

Figure 3a shows the XRD patterns of HNT, LDH and HNT@LDH in powder form mixed with grapefruit seed extract and compared with the pattern of pure grapefruit oil. The diffractograms of HNT and LDH show no significant changes after the incorporation of the oil. The (003) reflection of LDH, in both LDH + GO and HNT@LDH + GO, remains positioned at around 2*θ* = 10(°), corresponding to an interlayer distance of 8.9 Å, related to the presence of nitrates in the interlayer region. The presence of GO in all three cases is indicated only by the broad band located at 2*θ* = 15–25(°). Figure 3b shows the XRD patterns of the alginate beads prepared with HNT, LDH and HNT@LDH previously mixed with GO. The three patterns are superimposable with that of beads, consisting of alginate beads and GO without any filler. No reflections attributed to HNT or LDH are distinguishable due to the predominance of the amorphous bands related to the polymer.

**Figure 4:**
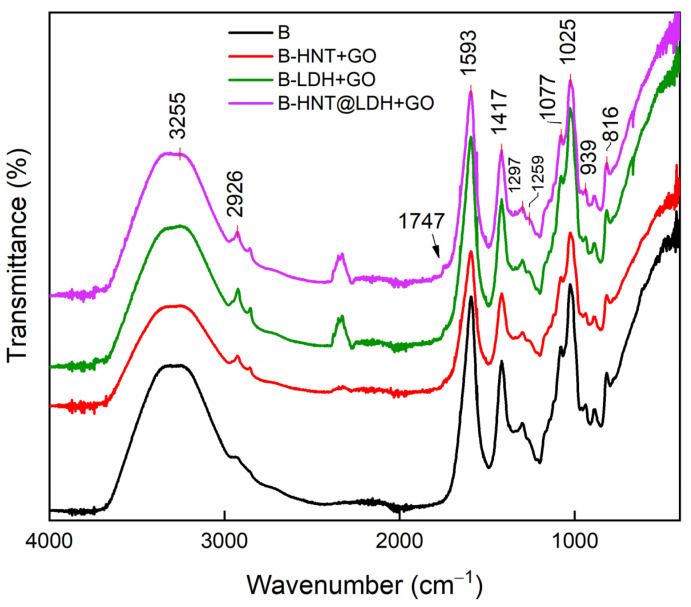
ATR spectra of alginate spheres containing GO and alginate spheres prepared with HNT, LDH and HNT@LDH mixed with GO.

Alginate signals are also predominant in the ATR spectra of the samples shown in Figure 4 and cover those referable to the inorganic fillers and GO. In particular, alginate is distinguished by the asymmetric and symmetric carboxyl stretching at 1593 and 1417 cm^−1^ and by the C-O-C stretching at 1025 cm^−1^. Grapefruit oil is represented by the shoulder at 1745 cm^−1^ attributable to C=O stretching of the linoleic carboxyl acid. This band is more intense in the sample containing HNT@LDH as a filler.

### 3.3. SEM/EDX Analysis

**Figure 5:**
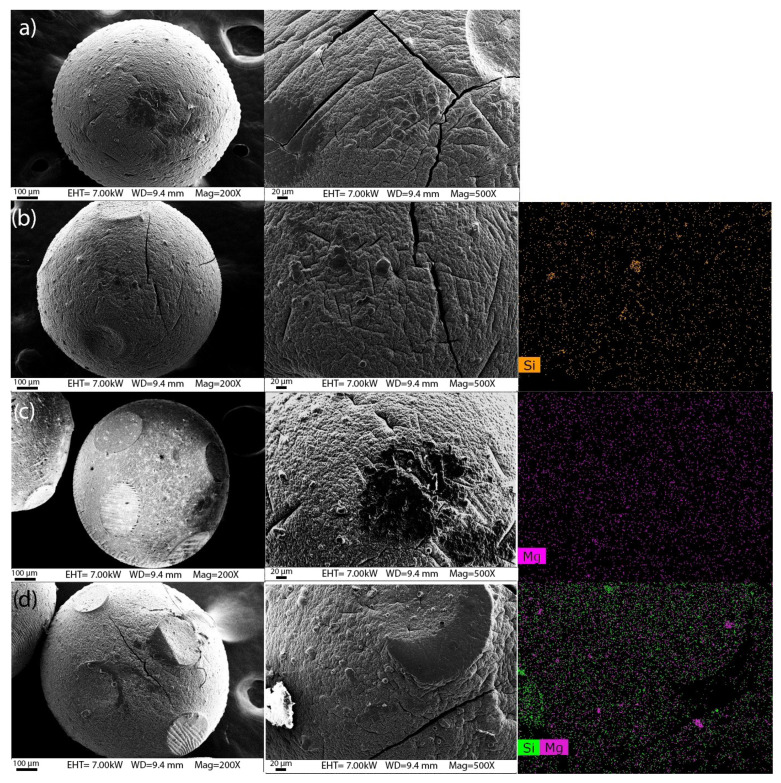
SEM images and EDS mappings of the spheres of alginate and GO (**a**) (scale bar = 100 µm), alginate and HNT + GO (**b**) (scale bar = 20 µm), alginate and LDH + GO (**c**) and alginate and HNT@LDH + GO (**d**).

The morphology of pristine HNT and HNT@LDH was investigated using SEM. Figure S1 shows HNTs with a smooth surface and length ranging from 200 to 1000 nm. After the precipitation of LDH, the surface of the nanotubes appears fully covered with nanocrystals with an average diameter of 20 nm. SEM-EDS images of alginate microbeads, prepared with and without filler and loaded with GO, are shown in Figure 5. All beads have a spherical morphology and a granular and compact surface. The size ranges between 770 and 920 μm. The surface appears rougher in the beads containing LDH + GO and HNT@LDH + GO (Figure 5c,d and Figures S2 and S3). Elemental mapping of the surfaces allows us to verify the degree of distribution of each filler within the matrix. The presence of HNT and LDH is indicated by the elemental distribution of Si and Mg, respectively. From the comparison of the EDS mappings of beads containing HNT + GO and HNT@LDH + GO versus those prepared with LDH + GO alone, it is clear that HNT + GO is more evenly distributed and better retained in the alginate microsphere: in the sample prepared with LDH + GO alone, the Mg content is very low. In contrast, when LDH is anchored on HNT, as in the sample in Figure 5d, the content of Mg increases. It is, therefore, likely that HNT facilitates the compatibility of LDH with alginate.

Table 2 reports the mean diameter of alginate beads from digital image analysis. According to the reported data, there are no noticeable differences between the different samples so the introduction of nanohybrids cannot affect the dimensions of the beads.

The GO loading evaluation highlights that the HNT + GO system is able to load about 64%. In addition, the loading amount decreased for LDH carriers while an intermediate value was obtained for HNT@LDH.

### 3.4. Thermogravimetric Curves

**Figure 6:**
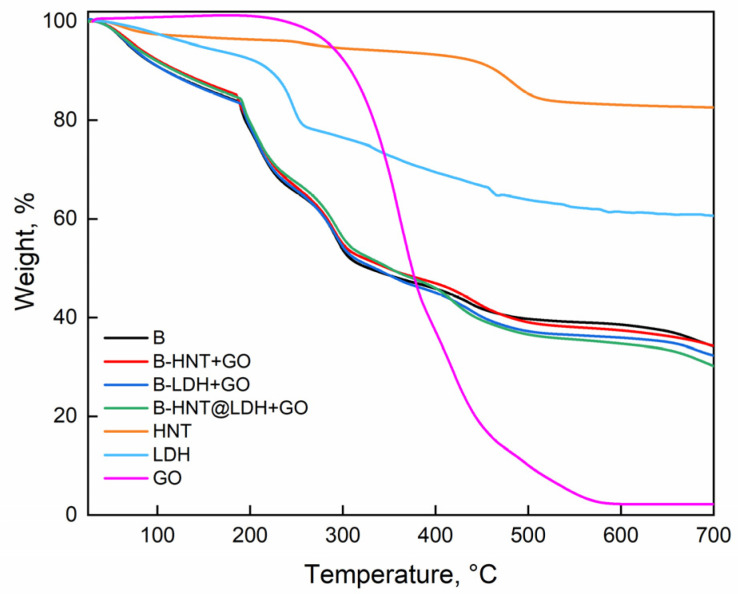
Thermal analysis of pure alginate beads, B-HNT + GO, B-LDH + GO and B-HNT@LDH-GO.

Figure 6 illustrates the weight (%) of pure beads and beads filled with LDH, HNT and HNT@LDH loaded with GO as a function of temperature (°C).

At temperatures below 200 °C, physically adsorbed water and interlayer water is desorbed from all the beads. The TGA of alginate beads shows an initial weight loss of approximately 16% wt due to the evaporation of water. The mass loss between 200 and 500 °C is attributed to the breaking of the alginate backbone and loss of hydroxyl groups caused by dehydration [48,49,50,51]. Above 300 °C, the polymer decarboxylates, mainly forming carbon dioxide and volatile low-molecular-weight compounds. The decomposition of grapefruit seed oil occurs over a wide temperature range (from 280 up to 590 °C) and is associated with the degradation of saturated and unsaturated fatty acids in the oil [52]. The mass loss of halloysite nanotubes is characterized by a single mass-loss stage at around 500 °C corresponding to the dehydroxylation of aluminol groups. The decomposition steps of LDH involve the loss of intercalated water (150 °C) and thermal decomposition of interlayer anions, followed by the transformation of the hydroxide framework into the corresponding oxide via dihydroxylation [53]. As for the alginate composite beads, they display the thermal events characteristic of HNT, LDH, GO and sodium alginate. The onset temperature for the main thermal events of the composite beads was found to be similar to that of the neat alginate bead. No noticeable difference in thermal stability was observed for alginate composites.

### 3.5. Release Kinetic Studies

**Figure 7:**
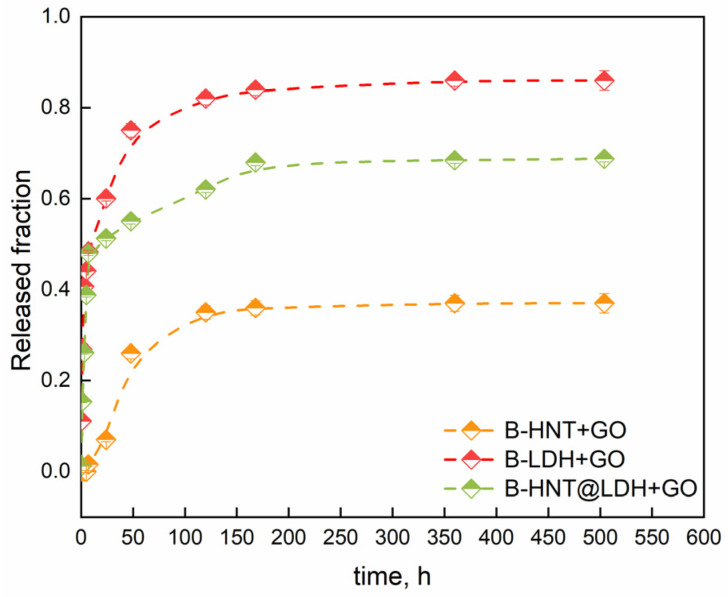
Release profiles of linoleic acid of GO from alginate beads.

To assess the effectiveness of alginate beads as a controlled-release system, we monitored the release of linoleic acid, one of the major components in the oil mixture, using a UV-vis spectrophotometer (Figure 7).

**Figure 8:**
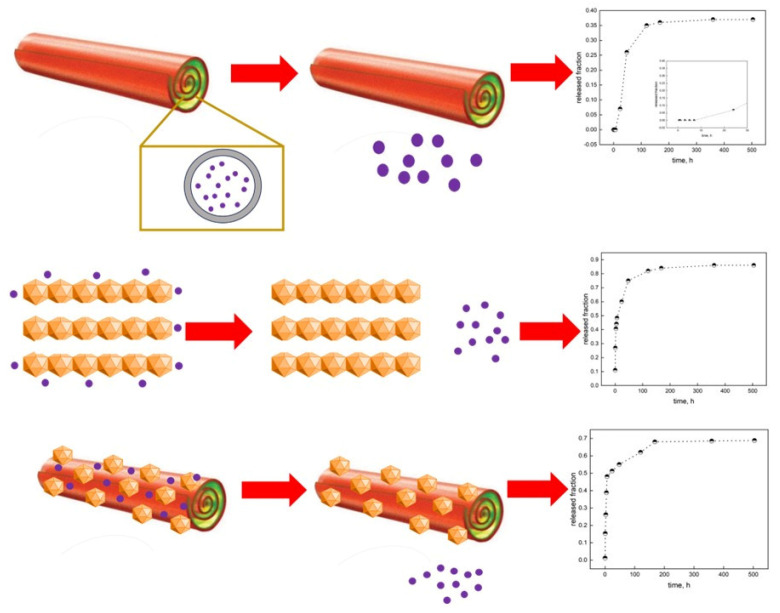
Schematization of the GO release from HNT, LDH and HNT@LDH.

The evaluation of the release kinetics is fundamental for understanding the delivery processes and tailoring the release according to the desired outcome. Both B-LDH + GO and B-HNT@LDH + GO exhibited a strong initial burst release within the first hours (39 and 45%, respectively). This may be due to the rapid diffusion from the LDH surface through the pores of alginate beads. The release rate slows down until it reaches a plateau after approximately 170 h. The increase in release rate during the first 24 h could be associated with the swelling of the alginate matrix and the osmotic pressure between the release medium and the bead. Water molecules are believed to induce polymer chain relaxation and speed up the release rate. In the case of B-HNT + GO, the release is notably slower and more controlled, which could be attributed to an enhanced mass transfer resistance offered by the intercalation of GO inside the HNT lumen. This phenomenon might be due to the unique structure of the HNT, which has a tubular morphology resulting from the rolling-up of the layers. An initial induction was observed before the first step concerning the burst release, which occurred at a slower rate. The first step is related to the diffusion of free oil molecules and molecules attached to the HNT surface. The equilibrium stage was reached after 118 h. The release profile reflects the localization of GO within the HNT cavity and the LDH surface. It should be noted that the incorporation into the HNT@LDH free spaces generates a sustained-release profile due to confinement and tortuosity effects. The low equilibrium-released amount proves that the oil was strongly incorporated within HNTs. Moreover, the presence of LDH on the surface of HNTs increases the specific surface area of the nanotubes and creates additional cavities/spaces in which GO can be trapped. In a previous study, it was found that the specific surface area of the HNT@LDH nanocomposite is approximately 22% higher than that of the pure HNT; furthermore, the synthesis process affects the lumen of the nanotubes [34]. The strong alkaline synthesis condition results in partial dissolution of the inner concave surface of the HNT, leading to an increase of about 14 nm in the diameter of the lumen. The wider pore diameter is beneficial for the incorporation of large molecules, like those found in essential oils, within the lumen. The release of GO from different structures has been schematized in Figure 8.

### 3.6. Radical Scavenging Activity

**Figure 9:**
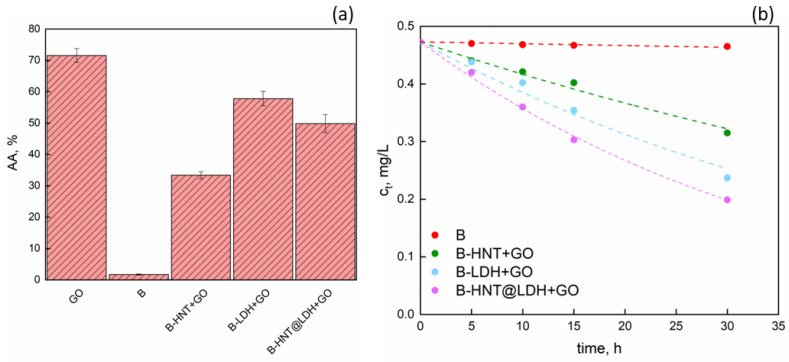
(**a**) Radical scavenging activity (%) of GO, B, B-HNT + GO, B-LDH + GO and B-HNT@LDH + GO, and (**b**) fitting curves of *c_t_* data using Equation (4).

The DPPH scavenging assay was used to determine the free radical scavenging ability of alginate beads (Figure 9).

GO demonstrated to be a potent antioxidant, showing a radical scavenging activity of 72%, which can mainly be attributed to the presence of active substances [54]. The antioxidant activity of GO is well-known, and its activity is mainly derived from polyphenols such as flavonoids, tocopherols and other bioactive functional components present in GO [55]. Since anthocyanins are rich in phenolic hydroxyls, the antioxidant activity of GO is mainly related to phenolic hydroxy [56]. In addition, the neat alginate beads showed negligible AA, while the presence of GO led to noticeable AA, with % values of 33.4, 49.8 and 57.9% for B-HNT + GO, B-HNT@LDH + GO and B-LDH + GO, respectively. The effective intercalation of GO into HNTs and LDH can prevent free interaction with oxidizing free radicals.

The radical scavenging data were modelled through an *n*-th order model (Equation (3)):

(3) $$-\frac{dc_{t}}{dt}=k{c_{t}}^{n}$$

where *c*_0_ is the initial concentration of DPPH, *c_t_* is the time-dependent concentration and *k* is the reaction constant. Integrating Equation (3), the following expression can be obtained (Equation (4)):

(4) $$c_{t}^{1-n}-c_{0}^{1-n}=\left(n-1\right)*k*t$$

A nonlinear fitting procedure was applied to evaluate the parameter *n* and the rate constant *k*. The larger the rate constant *k*, the faster the corresponding free radical scavenging rate and the higher the antioxidant capacity. The *k* and *n* values are reported in Table 3.

As can be observed from Table 3, the radical scavenging activity is relatively low for pure alginate beads while the rate constants increased in the following order: B-HNT + GO > B-LDH + GO > B-HNT@LDH + GO. The addition of GO to the beads significantly increased the DPPH radical clearance of the alginate beads. The parameter *n* approaches the value *n* = 1 and decreases according to the order B-HNT@LDH + GO < B-LDH + GO < B-HNT + GO < B.

These data are perfectly in agreement with the release profiles reported in Figure 7.

## 4. Conclusions

In this study, a novel antimicrobial release system based on alginate hydrogel filled with nanotubes of halloysite (HNTs), LDH and flower-like structure grown on HNTs (HNT@LDH) was fabricated. Grapefruit seed oil (GO), as a mixture of antimicrobial compounds, was impregnated into inorganic nanofillers through a vacuum-assisted process. Ionotropic gelation was applied to produce the hybrid systems. The beads were characterized by carrying out XRD and FTIR analyses. The spherical morphology of beads, and the homogenous distribution of Si and Mg, were evaluated using SEM. Finally, the radical scavenging ability of beads was demonstrated through a DPPH-based methodology. The neat alginate beads showed negligible antioxidant activity, while the presence of GO led to 33.4, 49.8 and 57.9% activity for B-HNT + GO, B-LDH + GO and B-HNT@LDH + GO, respectively. The release kinetics appeared to be different and related to the properties of the inorganic materials. The release profile reflects the presence of GO within the HNT cavity and the LDH surface, while the incorporation into the HNT@LDH free spaces generates a sustained-release profile due to confinement and tortuosity effects.

The effective intercalation of GO into HNT and LDH can prevent free interaction with oxidizing free radicals. It follows that the obtained systems are very promising in the bio-packaging field.

## Figures and Tables

**Table 1:** Grapefruit seed oil composition.

Peak Number	Compound	% wt
1	Lauric acid	0.68
2	Tetradecanoic acid	1.56
3	Palmitic acid	36.17
4	Octadecanoic acid	2.38
5	Oleic acid	27.81
6	Linoleic acid	27.38
7	Linolenic acid	2.51
Other compounds		1.51

**Table 2:** Mean diameter of alginate beads and GO loadings.

Sample	Diameter, mm	GO Loading, %
B	0.87 ± 0.10	-
B-HNT + GO	0.88 ± 0.22	64
B-LDH + GO	0.77 ± 0.09	47
B-HNT@LDH + GO	0.92 ± 0.13	52

**Table 3:** Values of *k* and *n* obtained from Equation (4).

Sample	*n*	*k*, 1/min	R^2^
B	1.05	0.0007	0.991
B-HNT + GO	0.93	0.0115	0.985
B-LDH + GO	0.92	0.0192	0.989
B-HNT@LDH + GO	0.89	0.0254	0.997

## Data Availability

Data are contained within the article and Supplementary Materials.

## Supplementary Materials

The following supporting information can be downloaded at: https://www.mdpi.com/article/10.3390/nano14020232/s1, Figure S1: SEM images of pristine HNTs and HNT@LDH (scale bar = 100 nm); Figure S2: Profile plots of the surfaces of pure alginate beads (black) and alginate beads with HNT (green), LDH (cyan) and HNT@LDH (magenta); Figure S3: Surface plots of the surfaces of pure alginate beads and alginate beads with HNT, LDH and HNT@LDH.
