# Supplementary material for: Alginate Microbeads Containing Halloysite and Layered Double Hydroxide as Efficient Carriers of Natural Antimicrobials
Source: Nanomaterials (Basel). 2024 Jan 21;14(2):232. doi: 10.3390/nano14020232 (PMC10820769; doi:10.3390/nano14020232)
Supplement: Supplementary file 1 [file nanomaterials-14-00232-s001.zip › nanomaterials-2811423-supplementary.pdf]

nanomaterials

Supplementary Materials

Alginate Microbeads Containing Halloysite and Layered Double Hydroxide as Efficient Carriers of Natural Antimicrobials

Gianluca Viscusi [1], Elisa Boccalon [2], Elena Lamberti [1], Morena Nocchetti [3] and Giuliana Gorrasi [1,*]

[1] Department of Industrial Engineering, University of Salerno, Via Giovanni Paolo II 132, 84084 Fisciano (SA), Italy; gviscusi@unisa.it, ellamberti@unisa.it , ggorrasi@unisa.it
[2] Department of Chemistry, Biology and Biotechnology, University of Perugia, Via dell' Elce di Sotto 8, 06123 Perugia (PG), Italy; elisa.boccalon@gmail.com
[3] Department of Pharmaceutical Sciences, University of Perugia, Via del Liceo 1, 06123 Perugia (PG), Italy; morena.nocchetti@unipg.it

* Correspondence: ggorrasi@unisa.it

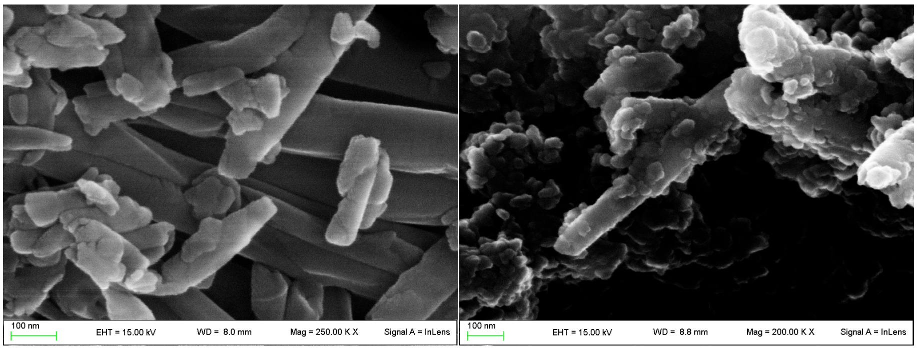

Figure S1. SEM images of pristine HNT and HNT@LDH (scale bar=100 nm).

The pictures of the samples were processed using ImageJ software to highlight the varying surface texture. Firstly, the pictures were converted into 8-bit images. Then, a rectangular area of the same size was selected for each picture, located in the flattest part of the surface of the alginate spheres. Finally, a profile plot was created to display the intensities of the grey scale of the surface. The experiment was repeated by plotting the entire surface covered by the rectangle. Figures S2 and S3 display the profile and surface plots of the beads, respectively. In the profile plot of alginate beads, the variation of the intensity of the grayscale is smaller when compared to the profile plot of simple alginate beads and alginate beads with filler. The same difference is displayed by the three-dimensional graphs of the intensities of pixels in the grayscale or pseudo-colour image. The mentioned differences indicate that there is an increase in roughness in the texture of composite beads.

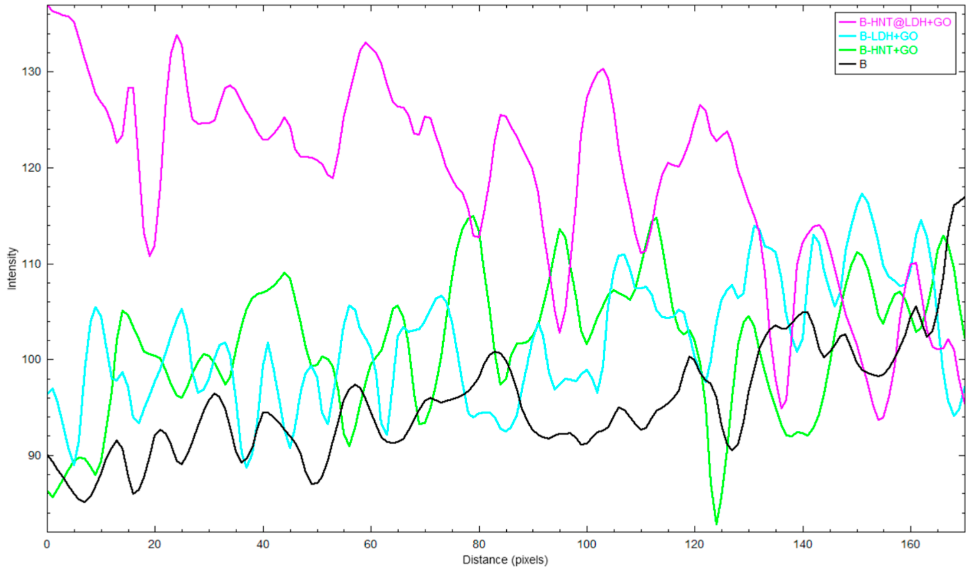

Figure S2. Profile plots of the surfaces of pure alginate beads (black), and alginate beads with HNT (green), LDH (cyan), and HNT@LDH (magenta).

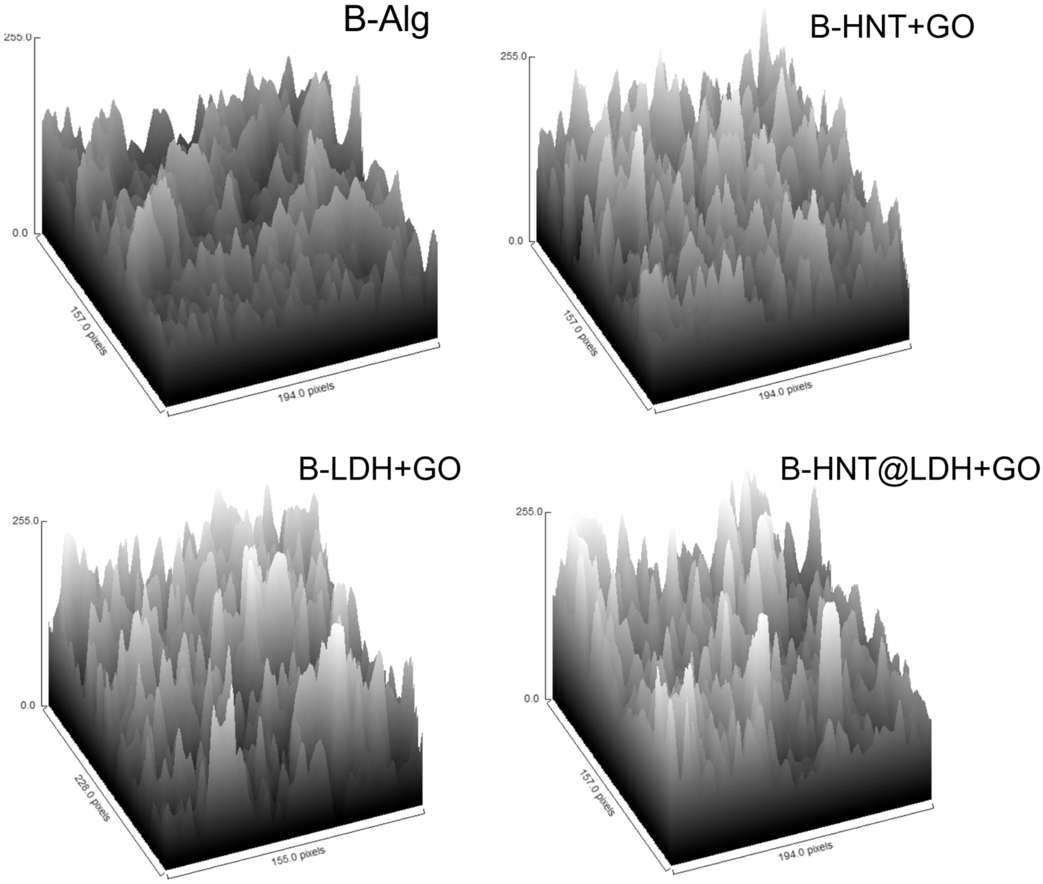

Figure S3. Surface plots of the surfaces of pure alginate beads, and alginate beads with HNT, LDH, and HNT@LDH.
